# Design and Implementation of a Cloud Computing Adoption Decision Tool: Generating a Cloud Road

**DOI:** 10.1371/journal.pone.0134563

**Published:** 2015-07-31

**Authors:** Iñaki Bildosola, Rosa Río-Belver, Ernesto Cilleruelo, Gaizka Garechana

**Affiliations:** 1 Foresight, Technology and Management (FTM) Group, Industrial Organization and Management Engineering Department, Faculty of Engineering in Bilbao (ETSI Bilbao), University of the Basque Country UPV/EHU, Basque Country, Spain; 2 Foresight, Technology and Management (FTM) Group, Industrial Organization and Management Engineering Department, University College of Engineering of Vitoria-Gasteiz, University of the Basque Country UPV/EHU, Basque Country, Spain; 3 Foresight, Technology and Management (FTM) Group, Industrial Organization and Management Engineering Department, Escuela Universitaria de Estudios Empresariales de Bilbao, University of the Basque Country UPV/EHU, Basque Country, Spain; University of Cape Town, SOUTH AFRICA

## Abstract

Migrating to cloud computing is one of the current enterprise challenges. This technology provides a new paradigm based on “on-demand payment” for information and communication technologies. In this sense, the small and medium enterprise is supposed to be the most interested, since initial investments are avoided and the technology allows gradual implementation. However, even if the characteristics and capacities have been widely discussed, entry into the cloud is still lacking in terms of practical, real frameworks. This paper aims at filling this gap, presenting a real tool already implemented and tested, which can be used as a cloud computing adoption decision tool. This tool uses diagnosis based on specific questions to gather the required information and subsequently provide the user with valuable information to deploy the business within the cloud, specifically in the form of Software as a Service (SaaS) solutions. This information allows the decision makers to generate their particular Cloud Road. A pilot study has been carried out with enterprises at a local level with a two-fold objective: to ascertain the degree of knowledge on cloud computing and to identify the most interesting business areas and their related tools for this technology. As expected, the results show high interest and low knowledge on this subject and the tool presented aims to readdress this mismatch, insofar as possible.

## Introduction

Although there is no general definition of cloud computing [1], [2], one of the most used definitions is from the National Institute of Standards and Technology (NIST) which states: "cloud computing is a model for enabling ubiquitous, convenient, on-demand network access to a shared pool of configurable computing resources that can be rapidly provisioned and released with minimal management effort or service provider interaction". Its most important features can be summarized as follows: on-demand self-service, broad network access, resource pooling, rapid elasticity or expansion, and measured service [3]. In cloud computing IT service providers offer their services grouped into three categories, Infrastructure as a Service (IaaS), allowing users to deploy hardware computing resources as a service, Platform as a Service (PaaS), providing all the necessary components for creating a new software application, and Software as a Service (SaaS), offering the consumer a wide variety of applications provided by service providers and running on the infrastructure of the cloud [4–7]. In addition, there are four deployment models: Private, the cloud infrastructure is operated solely for an organization; Community, the cloud infrastructure is shared by several organizations; Public, the cloud infrastructure is made available to the general public and is owned by an organization selling cloud services; Hybrid, The cloud infrastructure is a composition of two or more clouds (private, community, or public) [3].

A study conducted by the Cisco company [8] revealed the following impressive figures concerning the cloud computing impact sphere. Global cloud traffic:

- Annual global cloud IP traffic will reach 5.3 zettabytes (10^21 bytes) by the end of 2017. By 2017, global cloud IP traffic will reach 443 exabytes (10^18 bytes) per month (up from 98 exabytes per month in 2012)
- Global cloud IP traffic will increase nearly 4.5-fold over the next 5 years. Overall, cloud IP traffic will grow at a Compound annual growth rate of 35 percent from 2012 to 2017
- Global cloud IP traffic will account for more than two-thirds of total data center traffic by 2017

In addition, cloud computing is expected to be the main driving force behind IT sector development and SMEs will be the strategic market. Moreover, it is forecasted that 13.8 million jobs will be created over the 2011–2015 period [9].

North America represents the largest opportunity for SaaS, as well as being the most mature of the regional markets [10]. Furthermore, Gartner says that European cloud adoption will lag behind U.S. by at least two years. The main reasons for this statement are European privacy rules, multi-country business processes, a deep euro crisis and a lingering recession [11]. The introduction to cloud technology occurs in different ways and depends largely on company size, in the case of Spain, for example, 100 percent of large companies are expected to adopt it by 2015. However, only 69% of SMEs will implement it [12]. The largest increase occurs in SMEs, reaching 69% in 2015, up from 13.9% in 2011 [13]. Experts unanimously agree that the trend for large companies will be the hybrid model and all kinds of families (IaaS, PaaS and SaaS). Nevertheless, SMEs will focus on the public model and the demand will be for SaaS applications [14].

It should be pointed out that cloud computing goes hand in hand with three other cutting edge information technologies, namely: big data and analytics, social networking and mobile computing, which are creating the 3rd platform [15] also known as Big Services Era [16]. These technologies are deeply interrelated, as will be made clear when analyzing the test results in the fifth section of this work.

It should be highlighted that there is a lack of knowledge concerning what cloud computing is, as well as its most significant benefits. In particular, 54.9% of microenterprises and SMEs confess that they have no knowledge of cloud technology. This circumstance results in a loss of SME competitiveness, contrasted with the fact that this technology model is particularly beneficial for SMEs [17], [18]. Furthermore, the lack of knowledge regarding the benefits (63.1%) is stressed as the greatest obstacle to entry into the cloud [14].

Migrating to cloud computing is not an easy issue, all the more when there is no widely accepted standard for cloud services and a need to develop guidelines and criteria to help entities understand their requirements. Linked with that, decision support tools that aid decision makers to migrate IT systems to the cloud are also required [19]. In fact, as it is an adoption of an emerging technology, it is regarded as a management innovation challenge related with technological innovations, where managerial interventions in organizational routines will be needed in order to overcome the ineffectiveness of familiar practices within the organization for dealing with a new technology. In addition to which, introducing a new innovation can result in employee resistance, thus, senior decision-makers in organizations need to prepare employees for this new learning curve by providing training and communication in advance of cloud implementation. To this effect, there has to be an awareness of what is being introduced, specially focused on the benefits it will bring [20].

Several frameworks have been presented as guides to make decisions on cloud technology implementation. From theoretical frameworks to specific examples, a few of them have gone beyond the theoretical paper-based approach and have developed an interface for the decision makers, in an attempt to make the process more user friendly. This paper describes the process of making a cloud computing technology adoption decision tool and its assessment performed via a pilot study, conducted with different local companies at Basque Country autonomous community (Spain) level. It should be noted that the Basque Country’s industry structure is based on SMEs, in fact 93.4% of the companies have less than 10 employees whereas 5.5% have between 10 and 49. In addition to this, the community ranks number one in Spain in terms of investment in research and development as a percentage of GDP (2.1%). Ultimately, the utilization of the tool, i.e. carrying out the diagnosis, shall permit the users to initiate their own Cloud Road, which will allow implementation of cloud computing technologies in order to enhance the efficiency of their business strategies.

## Methods

### Related Work

Generally speaking, a commonly selected criterion when it comes to analyze the adoption of cloud computing is to analyze the corresponding return on investment (ROI). Though current surveys find cloud computing highly suitable for small and medium enterprises, a deeper analysis of the economic aspects of migration to cloud architecture can provide very valuable information. Thus, various types of cloud cost-benefit analysis have been reported [21–23]. An evaluation of IT infrastructures to support the decision of whether to move information systems into the cloud or not was attempted in Khajeh-Hosseini et al. [24], where a Cost Modeling tool is presented and evaluated using a case study of an organization that is considering migration of some of its IT systems to the cloud, analyzing the specific resource usage and the deployment options being used by a system. A more generic model, containing variables which should be applicable to any company, was presented in [25], where a ROI model is implemented, identifying the factors that need to be considered and returning a certain profitability valuation from the change. Here, the concept of “initial information” is considered as a scenario definition, based on the entity’s IT utilization level and managed data characteristics. The tool includes some intangible benefits to give a broader picture. No supporting software was developed for the tool.

A multi-criteria approach could also be followed, to give a more generalized point of view, in order to support the decision about implementing cloud computing. In this sense, the decision is based by taking issues such as cost, benefits, opportunities, goals or risks, among other things into account. Several approaches have been presented based on this concept, focused on the outsourcing of IT technologies; such as Wang and Yang [26] and Udo [27], where initial information was not considered and no software tool adaptable to different decision-making goals was developed. A multi-criteria decision tool is presented in Menzel et al. [28], where a customized evaluation method is developed, and where generic multi-criteria frameworks are adapted to IT infrastructure solutions and more specifically to cloud computing, to be used as a decision tool. The evaluation method is selected and configured based on specific criteria and requirements in order to perform the evaluation itself, all this supported by a user interface via web application.

A more practical standpoint is provided by some web based initiatives. For instance, Cloud Harmony [29] is presented as a benchmarking tool, where a set of benchmarks is provided in order to objectively compare different cloud providers, analyzing the corresponding IaaS or PaaS provided. The benchmarks rank cloud providers accordingly with their network throughput, latency, performance (CPU, disk I/O and others) and service availability. With Cloud Sleuth [30], best practices and resources for deploying and managing applications in the cloud are provided, as well as applications to compare the performance and availability of PaaS and IaaS providers. The main approach of this kind of tool is not to provide a framework per se to the decision maker but as much information as possible.

Rather than focusing on the IT technology itself, several studies have focused on a more practical point of view, analyzing SaaS implementation in order to enhance business-specific processes. As a new concept, enterprise resource planning (ERP) software that is deployed into a cloud environment becomes “Cloud ERP Software” [31], with the addition of the fact that different business tasks could be migrated independently, so tailoring needs is perfectly feasible. To this effect, studies like Gerhardter and Ortner [32] try to analyze the success factors for SaaS implementation. These factors are determined by studying those which were successful from a classical ERP implementation and then by reconceiving them with the new cloud scenario.

Several frameworks have been presented when it comes to decide whether to adopt cloud computing or not and how to do it, based on business models or specific business processes. This concept is developed in Chang et al. [33], where an already proven theoretical framework called “Cloud Computing Business Framework” is described. Here the relation between the business model and the IT services is examined, and cloud adoption is presented as a project itself, identifying four key factors, namely “Business Model Classification” where the importance of the business model is stressed; “Organizational Sustainability Modeling” where an ROI is included; “Service Portability” from desktop to Cloud and between Clouds, and “Linkage” where linkage between different cloud services, and between business and services is explained. Some studies have been carried out in order to specify an approach based on one specific business activity, such as the “supply chain” approach in Wu et al. [34], where the circumstances that affect a firm’s intention to adopt cloud computing technologies in support of its supply chain operations are investigated, and a conceptual framework model is presented.

Finally it should be stated that few works have made the effort to clarify those variables that work as drivers (or barriers) when it comes to cloud computing adoption. Shimba F in [35], conducted a survey among several workers of both technical and non-technical job-roles and organizations in an attempt to identify these drivers and barriers. In the work he states that the biggest driving force for cloud adoption for most organizations was the need for flexible and scalable IT resources, followed by the need for resource optimization. Accordingly, costs were not the biggest driving force by themselves. However, Iyer EK et al. [36] reached a different conclusion in a later work which also aimed defining such drivers. Here the four gain vectors in the context of potential SME cloud adopters are provided based on a Delphi method process with industry experts, and cost saving gains take 80% of the perceived gain spectrum. Providing different reasons in order to explain this dominance, the paper states that perhaps the cloud computing industry (in terms of its communication to the market) has emphasized the cost gain element more than other non-monetizable ones. In addition to this, it is argued that the SME industry is, to a certain extent, always strapped for cash, and it can be safely surmised that any cost related benefit will tend to override other considerations.

Regarding this fact, it can be concluded that the potential initial drivers for cloud adoption, linked with its technological advantages, have been replaced for those directly related with cost savings, from a clear business approach. Linked to this, Marston et al. in [37] states that while the researchers and practitioners in the computer science community are making rapid strides in realizing cloud computing advantages in technological terms, an equally important discussion needs to start from a business perspective. Thus, our research aims at contributing increased knowledge in that perspective and, accordingly, the proposed model which is described in the next section follows this cost saving issue approach, based on the company perspective through its main strategies of competitive improvement, via general-purpose tools and SaaS recommendations.

### Proposed cloud adoption decision tool

Most of the existing approaches are based on a theoretical framework to aid the decision makers when adopting cloud computing. In fact, few of them have developed real tools. The provided information mainly comes from the analysis of adopting cloud computing or not, and in affirmative cases the migration of the already implemented IT technology is proposed. However, in order to obtain maximum practicality, our work aims to aid the decision makers when it comes to choose the most adequate software related to cloud computing, i.e. SaaS. In this sense, based on the recommendations given by the developed tool, the decision maker should be able to generate their own Cloud Road: the roadmap to implement cloud computing solutions in their company. Our work fills the existing gap from the explained approaches to the last step, the adoption of the cloud. This final step is no more than the SaaS selection, which is especially helpful for SMEs as discussed before. Furthermore, the developed framework is not only theoretical but a real tool which can be used several times in order to clarify the information and options provided.

**Fig 1:**
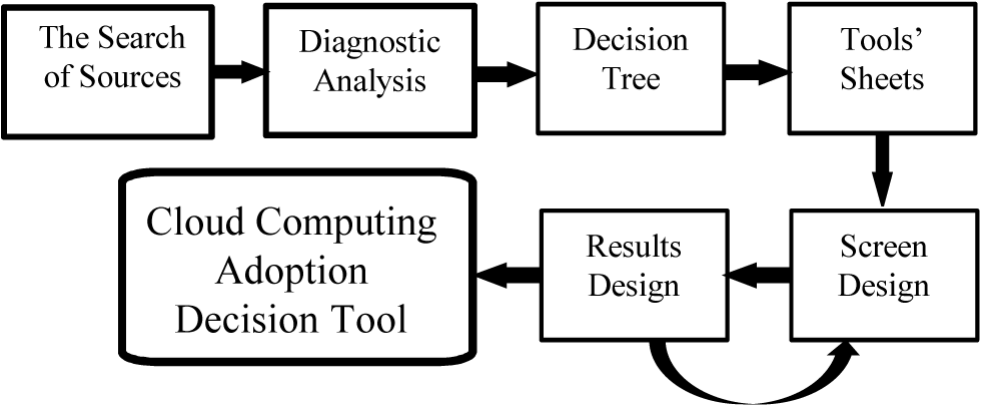
Research Methodology. The methodology used in the research is summarized in the figure, from the initial research to the achievement of the adoption decision tool.

The design process of a pilot tool was presented in Zabalza-Vivanco et al. [38], presented here is the final complete design, where all the functionalities and results are integrated. The methodology used in the research is summarized in Fig 1. The preparation phase of the decision tool is described in more detail below; this can be divided into two distinct stages: the development of the decision tree (S1 Fig) and its implementation.

#### Stage one: the creation of a decision tree

A decision tree is a diagram that shows conditions and actions in a sequential way [39]. Furthermore, this method allows us to show the relationship between each condition and the set of possible actions associated with it. Moreover, it provides a graphical view to aid the decision making process, indicating the variables that are assessed and what actions should be taken as well as the sequence in which decision-making must be executed.

The decision tree and consequently the designed decision tool is based on a diagnosis whose approach is enterprise level for the adoption of the cloud computing tools, mainly focused on SMEs. The goal of the tool is to identify the best actions for each company based on its strategy of competitive improvement. The model used is based on the postulates of Porter et al. [40], who described the competitive strategy as the offensive or defensive strategies of a company that create defendable positions within an industry.

Porter identified three generic strategies which could be used individually or combined in order to create a long-term defendable position: Cost leadership (consequently quality, service and cost reduction); Focus or Niche Strategy (focus on a particular buyer group, product segment, or geographical market); Differentiation (that offered by a firm means creating something that is perceived industry-wide as being unique). Later on in 1990 [41], he recognized the instability of those three generic strategies facing new market circumstances and the need of more dynamic models to design the competitive advantage. Following this approach, the competitive strategy of a given company must be constructed based on its competitive strengths and it should continue improving the most vulnerable areas. Thus, our decision tree and consequently the decision tool itself have been produced from a company perspective. In this sense, the company is questioned according to the main strategies of competitive improvement, consequently questions on offensive actions are particular to each company: the nature of the industry and its competitive position in terms of “company type”, “market/client diversification”, “product/service diversification”; together with the defensive actions where Porter’s generic strategy is followed in terms of cost leadership, hence the identification of which areas, resources and capacities the company wishes to improve is performed. For this purpose, “Clients”, “Government and Financial Institutions”, “Suppliers” and “HR” where included.

The complete decision tree is provided (separately due to its size), where the method of how to combine the aforementioned variables is shown. In this sense, the variables and their related tools are combined based on the priority levels set out by the company, which are the decision tree conditions: high, medium and low preference. For that purpose, an expert committee was created with members of all the agents involved in the project: staff from the Qmedia and Serikat companies; staff from the Tecnalia research center; and researchers from the Industrial Organization and Management Engineering Department of the University of the Basque Country. The main task of the committee was to evaluate each tool, generate a complete sheet for each and assign a grade to link them with the different variables; the whole process was conducted through several meetings until the final set was obtained. These sheets provide the information to generate the links within the decision tree and ultimately the combination of the variables themselves.

In order to select the proposed tools/SaaS within the decision tool consideration was given to the fact that business strategy was leading the way and accordingly those tools which can be used to increase the competitiveness of the company were prioritized. As a result, the approach taken is mainly based on Marston et al [37], which states that computing applications which are general-purpose in nature are the most appropriate to move to the cloud, since they offer tremendous economies of scale. Thus, based on the recommendations of that work, our decision tool follows this logic sequence when it comes to make recommendations: firstly general-purpose applications such as “office”, “email” and “collaboration”, applications with no specific requirements for any particular organization; after that standalone applications such as CRM, which are easy to deploy in a cloud; and lastly applications with intricate interdependence to the cloud. Several cloud applications available on the SaaS market were examined such as [42], taking into account all the aforementioned facts. The result was a set of 51 SaaS applications and tools which are grouped into different sections or functionalities. These functionalities are identified as: On-line Billing, On-line Accounting, Personal Management, Project Management, Backup and Storage, Document Management, Content Management System (CMS), Customer Relationship Management (CRM), e-Commerce, e-Mailing Marketing, Social Networking Management, Feeds Management, e-learning, Event Organization, Collaborative Platforms, Web Development, Video, Communication, Business Intelligence, Production Management, Warehouse Management, Diagrams, Enterprise Resource management (ERP), Quality Management, and Customer Service and Support.

#### Stage two: the development of the decision tool

The decision tool was implemented in C#, designed by a local company called Openaula which collaborated in the project with a design aimed at ease of use. It includes branching (conditions), templates and provides basic statistical analysis in order to process the results.

**Fig 2:**
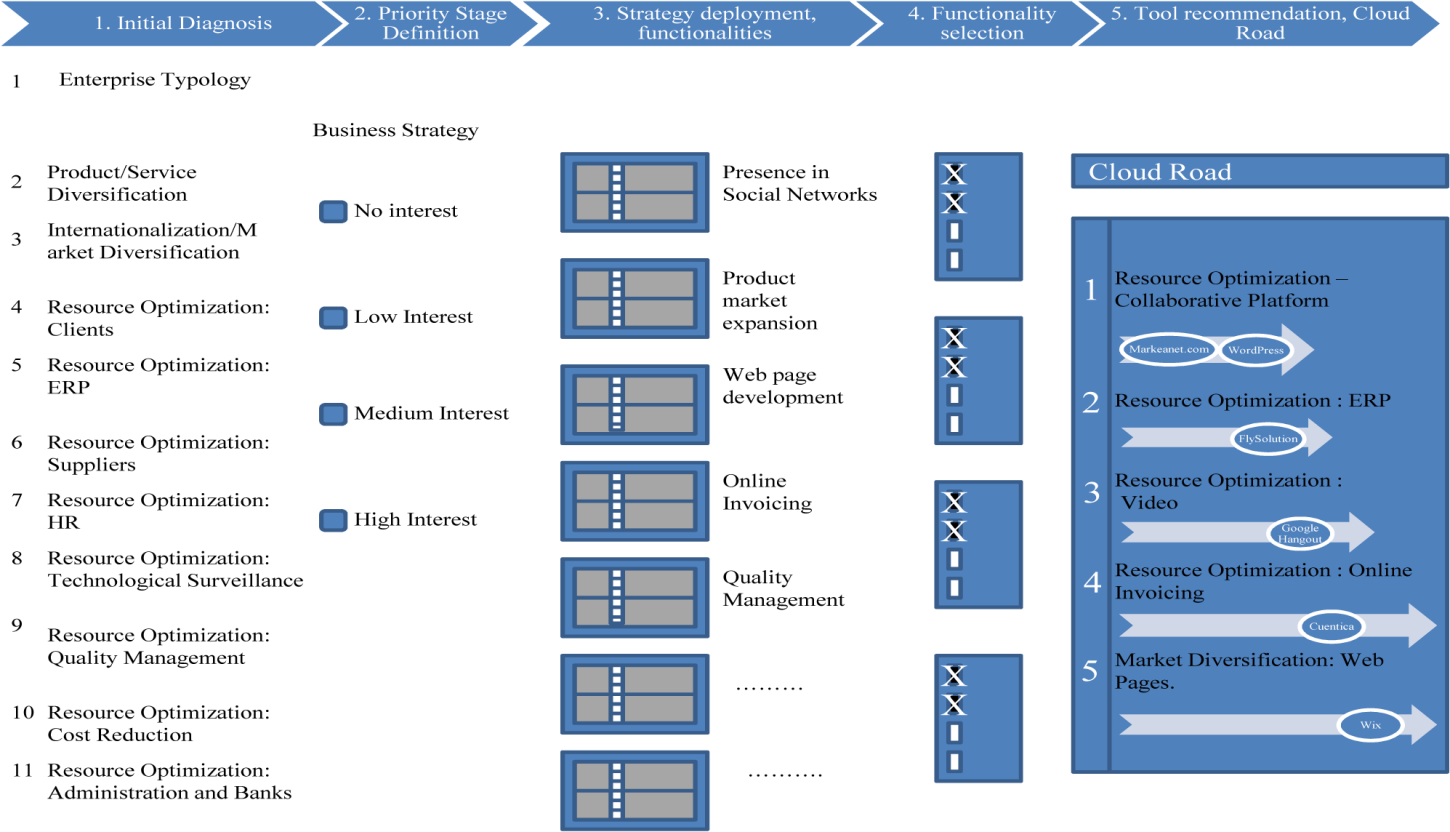
The decision tool operation: whole diagnosis and recommendation process. The whole process graphically represented, showing the different steps to be done by the tool user and the final recommendations to enhance the business areas and deploy the business strategy within the cloud.

**Fig 3:**
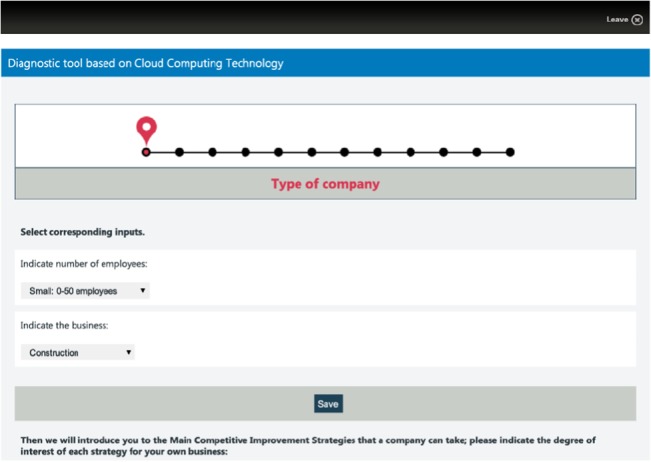
Step 1: Enterprise typology identification. The first step window capture, the tool gathers the user’s company size and sector.

**Fig 4:**
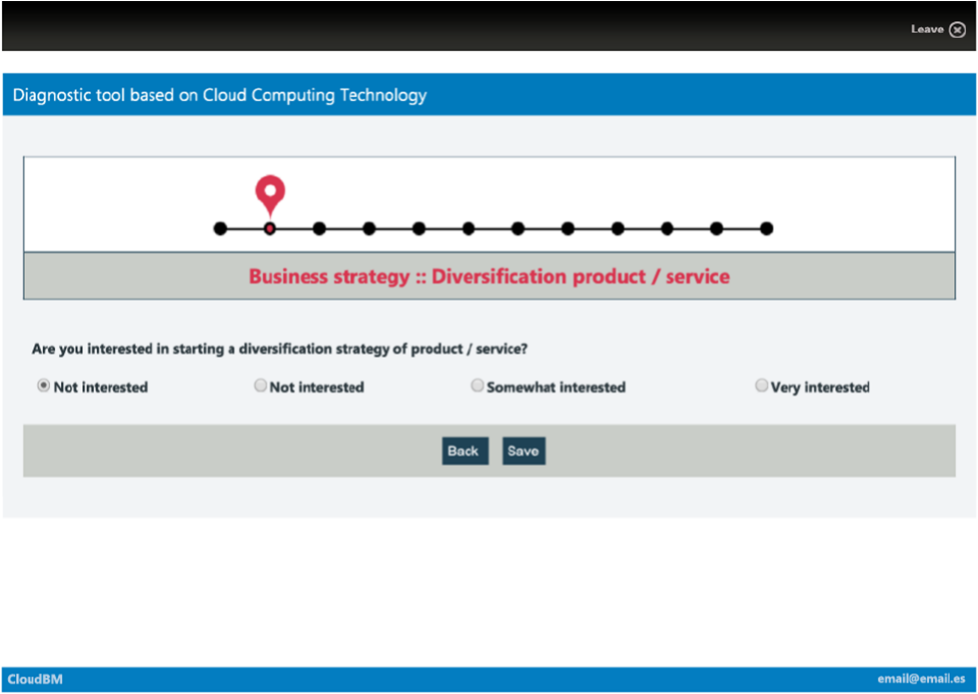
Step 2–11: Identification of the business area priority level. Standard window capture, steps 2–11 of the diagnosis, in which the user must indicate the priority level for each business area. The priority level goes from no interest to high interest.

**Fig 5:**
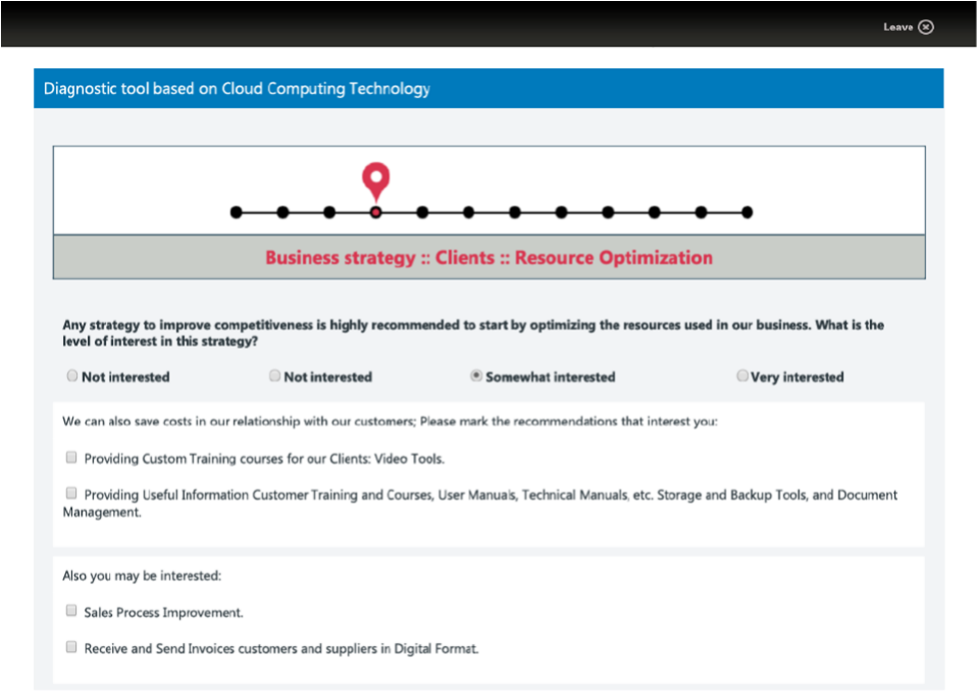
Step 2–11: Recommended functionalities and its selection to enhance business areas. Standard window capture, steps 2–11 of the diagnosis, in which the tool recommends several functionalities to enhance the corresponding business area, and the user selects the preferred ones.

**Fig 6:**
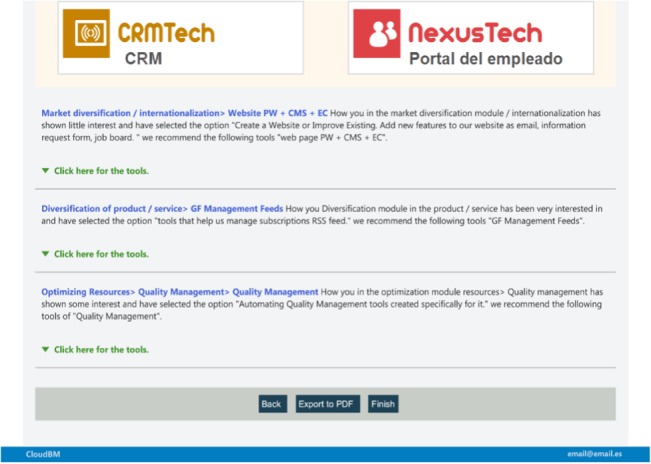
Recommendation of the decision tool to implement the selected functionalities. Final window capture, the decision tool offers and describes different solutions, basically in the form of SaaS, to enhance and deploy the business strategy within the cloud, based on the user’s previous selections.

The tool gathers information through eleven steps. Step 1 is used to obtain the size and sector of the entity. The following ten steps use the same procedure: the entity identifies ten different business areas with high, medium or low priority in order to improve the company situation by means of cloud computing adoption. In each step, some recommended functionalities will be shown which will improve those areas; the level of detail depends on the priority level. Finally, SaaS applications which allow the company to implement these actions will be suggested. The following captures (Figs 2–6) show the whole diagnosis process graphically, with different kinds of functionalities and tools provided to illustrate the diagnosis and recommendation process.

The resulting benefit of using this decision tool lies in the fact that, as a result, companies obtain a number of cloud solutions or tools that will enable them to improve their priority areas, generating a Cloud Road to implement cloud technology via SaaS. This is especially profitable for SMEs as discussed in the previous section, which are indeed the main target of this tool. Bear in mind that SMEs have reduced investment capacity and that the cloud’s business model, based on “on-demand service”, allows them to implement their priorities more tightly. Consequently, by means of this set of cloud SaaS solutions which fit the needs of each company, entities can reduce costs, improve the management of their customers, suppliers, employees and other agents such as public administration and banks. Furthermore, it is an aid for growth into new markets, as well as enhancing the task of creating new products or services. Note that on the final screen the basic features of each cloud tool are shown. In addition, clicking on any tool name, directs the user to the supplier’s webpage. Finally, it is worth pointing out that a tool ranking is generated: the different tools are ranked by the number of clicks received, which indicates how many times they have been visited. All these features are accessible in the tool: http://cloudbm.net/corporativa/default.aspx.

## Results and Discussion

### Methodology

**Fig 7:**
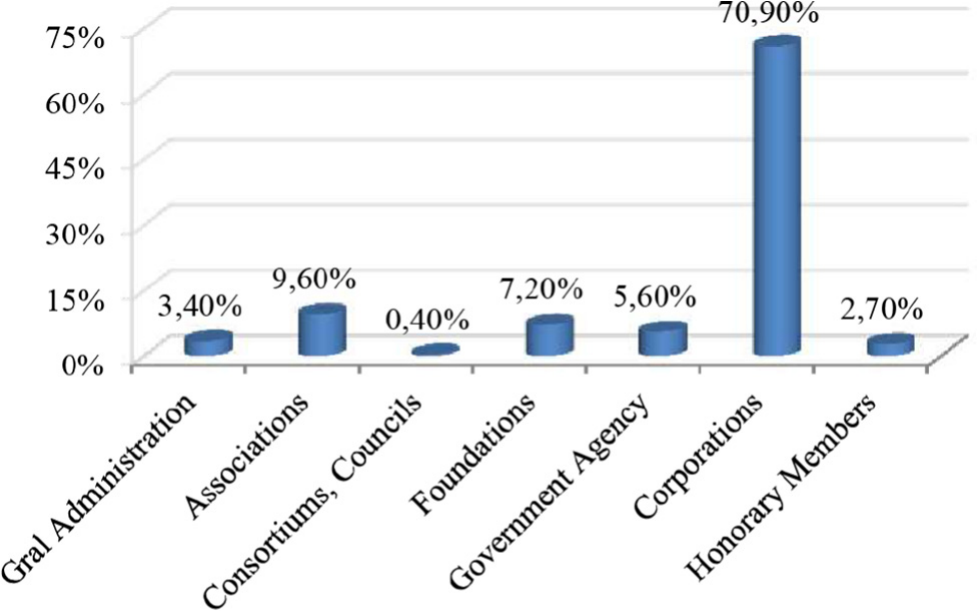
Profile of potential pilot study participants. The chart shows the profile of the potential participants of the pilot study using the tool.

In order to pursue a more objective assessment of our tool, a pilot study has been conducted among some SMEs in order to check which areas and features of the tool are more demanded and to check the overall behavior of the diagnosis. Accordingly, the most logical option was to conduct the study at the Basque Innovation Agency, named “Innobasque”, a private, nonprofit organization created to coordinate innovation in the Basque Country and to encourage entrepreneurial spirit and creativity. Innobasque membership is made up of all types of organizations, distributed as shown in the Fig 7. It has to be emphasized that 80% of the corporations are SMEs.

The method used to disseminate information about the tool was to mail all the members an introductory text describing it and providing the access link, supported by a news item published on the Innobasque website. This mailshot was sent on December 2013. In addition to this, once the project had been publicized, a forum was held at Innobasque’s premises in April 2014. The forum was used to directly present the tool itself: its functionalities, the diagnosis process and the results. The forum was attended by several enterprise decision makers and the pilot study was carried out. As a result of the pilot study 13 different companies completed the diagnosis satisfactorily, from which some provisional findings have been obtained, as will be seen in the following section. It should be noted that the participants validated the need of a tool to generate a cloud road and most of them reported having implemented the proposed tools. However, the tool is considered to be a live element and even though this first evaluation was centered on its design and usability, due to the continuous variability of the SaaS market, further results shall have to be gathered in order to evaluate the proposed tools and consider adding new solutions or removing some of the included.

### Pilot Study results

**Fig 8:**
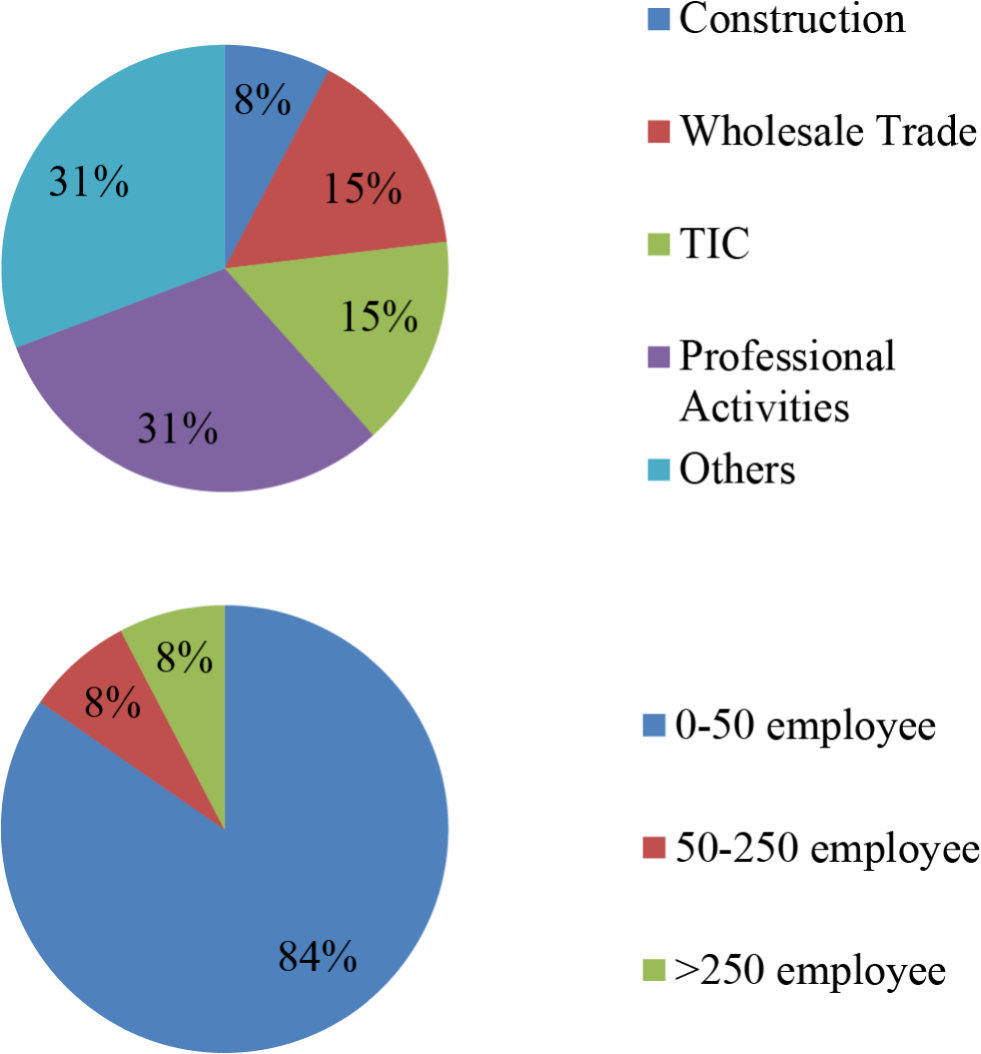
Typology of pilot study participants. The chart shows the typology of the final group of pilot study participants using the tool.

First of all, the tool gathers information regarding the enterprise’s sector and size, in order to generate a simple profile of each case. Different sectors are represented in the results since innovation does not clash with any specific sector and, as mentioned, sooner or later all enterprises will face cloud computing adoption. When it comes to size, most of the enterprises fall within the 0–50 employees category, whereas larger ones are less represented. It should be noted for future work that the 0–50 scale should be further subdivided, considering that most of the participants will be SMEs. The exact values are shown in Fig 8.

The analysis based on the participants’ answers is two-fold:

- On the one hand it is a discussion about business areas and priority levels.
- On the other hand the functionalities for each business area and the different solutions (i.e. SaaS) for those functionalities can be discussed.

**Fig 9:**
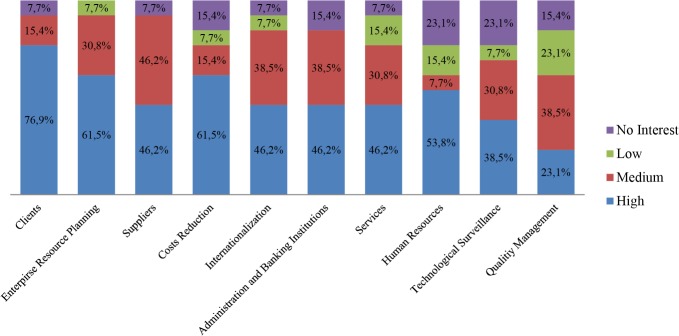
Participants’ interest level by business area. The chart shows the initial interest generated by each business area, among the pilot study participants.

Thus, when it comes to business areas, it is important to distinguish the participants’ preferences in terms of which business areas have attracted most interest. As a result, the tool asks the user to evaluate their interest in each of them by selecting from high, medium, low and no priority levels. There are 10 business areas reflected in the diagnosis and, as seen in Fig 9, not all of them obtain the same interest from the users.

The arrangement on the graph is performed by weighting the priority with 5, 3, 1 and 0 respectively from high to no interest. It is worth noting that the Clients business area has obtained more than 70% of high interest, along with ERP and Suppliers areas on the positive side of the balance, which obtain more than 80% of high and medium interest. At the other end lie the Human Resources and Quality Management areas which obtain 60% of that kind of interest. This may be due to the fact that cloud computing offers clear advantages when dealing with clients and ERP management in terms of ubiquity and flexibility, whereas Quality Management and probably Human Resources are not particularly benefited by the irruption of cloud computing. This has been well perceived by the participants. More surprising still is the poor ranking of Technology Surveillance, a clear cutting-edge business area apparently not yet well-known by firms within the study, but a must in the short-term.

**Fig 10:**
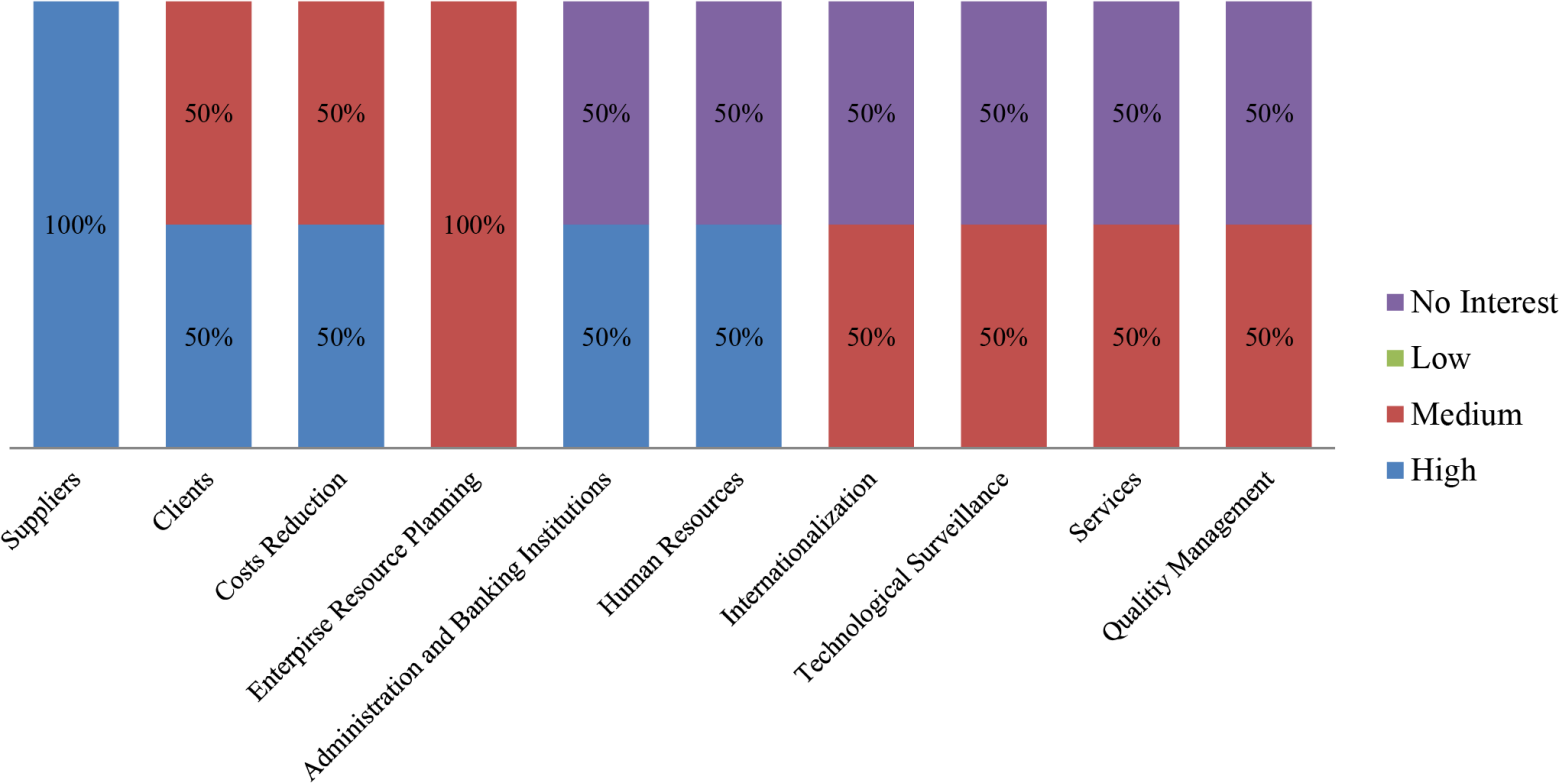
Participants’ interest level by business area. Wholesale sector companies. The chart shows the initial interest generated by each business area, among the pilot study participants pertaining to the wholesale sector.

**Fig 11:**
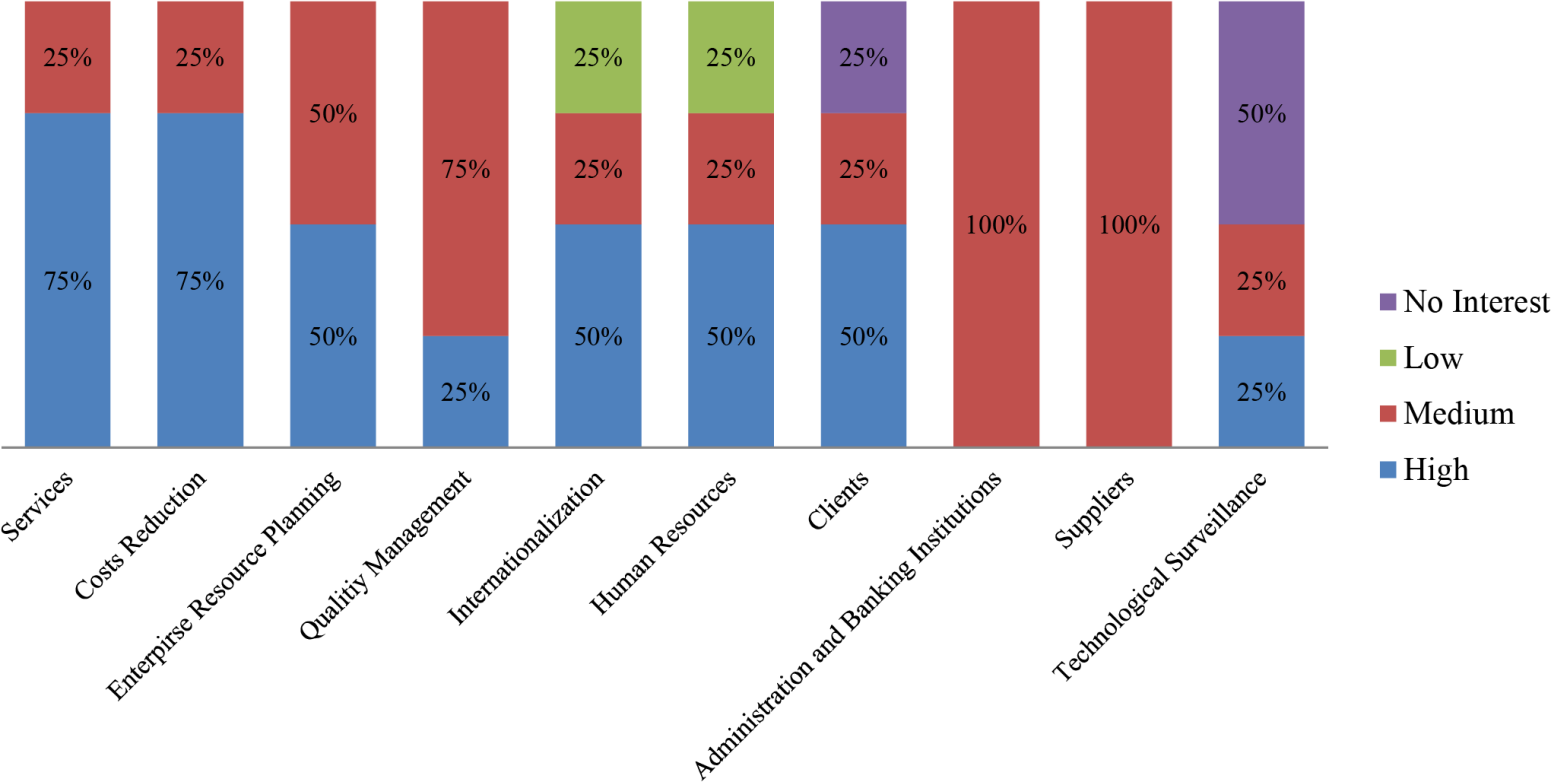
Participants’ interest level by business area. Professional activities sector companies. The chart shows the initial interest generated by each business area, among the pilot study participants pertaining to the professional activities sector.

**Fig 12:**
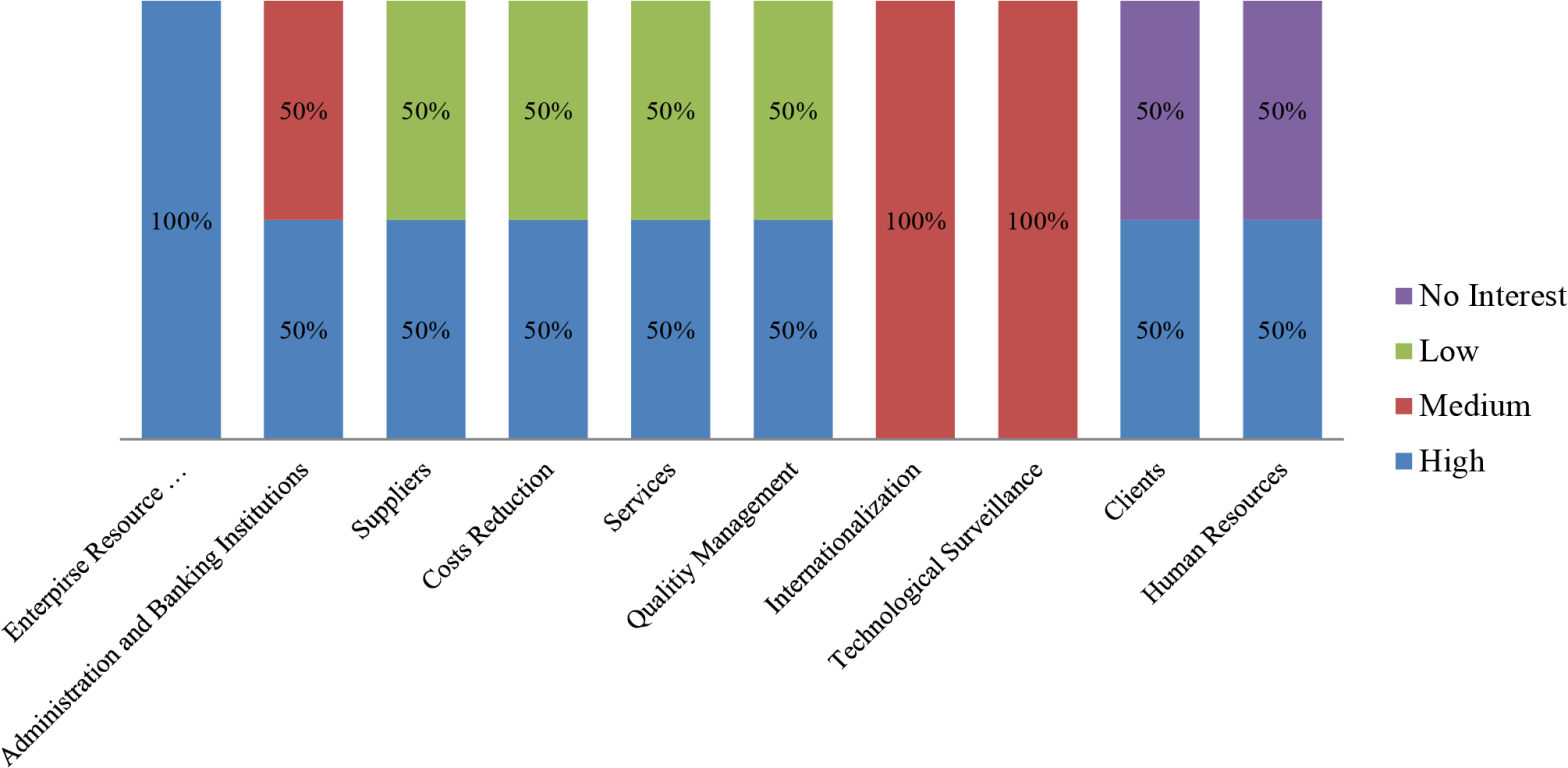
Participants’ interest level by business area. Information and communication technologies sector companies. The chart shows the initial interest generated by each business area, among the pilot study participants pertaining to the information and communication technologies sector.

**Fig 13:**
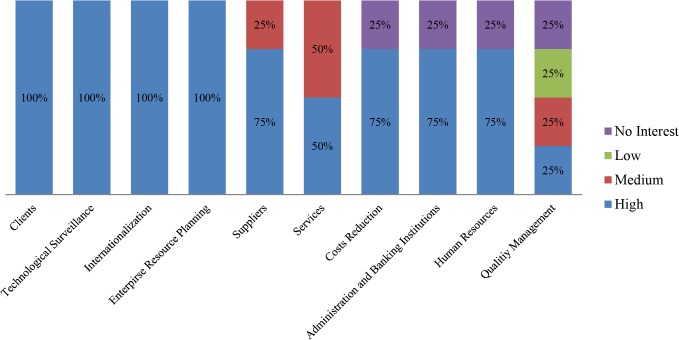
Participants’ interest level by business area. Other sector companies. The chart shows the initial interest generated by each business area, among the pilot study participants pertaining to other sectors.

In order to distinguish those interests but differentiating the enterprise sector, a deeper subdivision has been performed. Thus, the same information is offered, divided by business sector, in Figs 10–13.

The sample size does not permit any reliable conclusions to be drawn for each sector, however, the tool’s potential for the task is clear. Even if the ability to identify preferred business areas is valuable information when it comes to adopt cloud computing technology, a breakdown by enterprise sector offers a far more in-depth view of specific strategies. Thus, the results make it possible to conclude that even if the breakdown is performed, uniformity of preferences is not assured, as can be seen in the “Professional Activities” sector, where different kinds of priority levels are selected in most of the business areas. This inconsistency would be overcome when using larger sample sizes. Analyzing different sectors in order to discover similarities shall allow them to be grouped by their interest patterns revealing, at least in part, which areas of business should be exploited by cloud computing technology in each sector.

**Fig 14:**
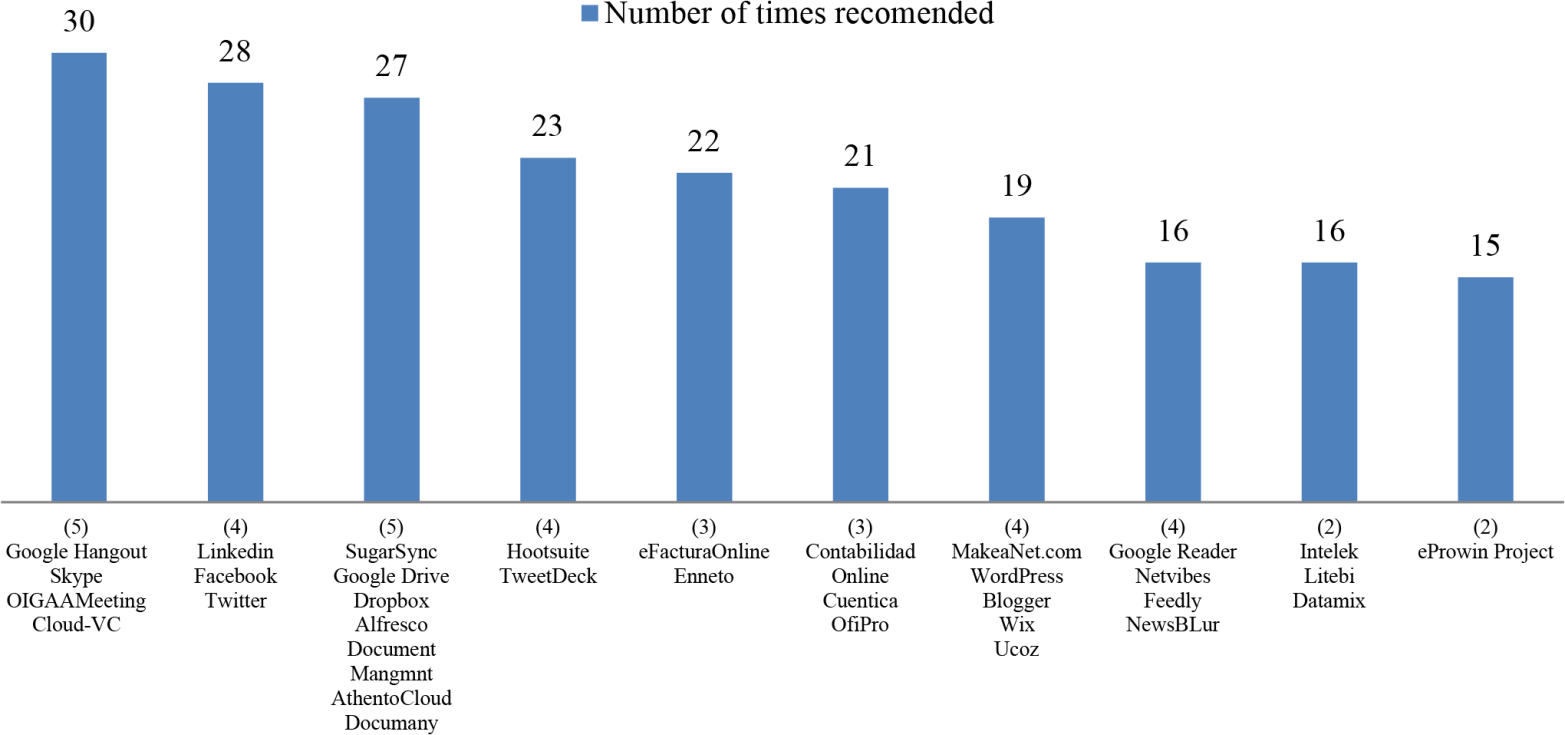
Top ten tools by number of times recommended. The chart shows the top ten tools, arranged by the total number of times recommended to the participants in the pilot study. In brackets the number of business areas in which each tool appears.

When it comes to analyze the functionalities of each business area and the solutions to those functionalities it should be noted that those solutions can be either a specific existing SaaS or a different kind of solution (i.e. a social network such as Facebook), so for the sake of clarity, both will be identified generically as tools. With regard to the functionalities and the tools analyzed in the diagnosis, the first interesting point is to show which specific tools have been recommended more frequently. Accordingly, Fig 14 shows the top ten tools based on how many times they have been proposed. It should be noted that some specific functionalities and the corresponding tools are included in different business areas, as it is the case, for instance, with the “Subscription to Social Networks” functionality and its specific proposed solutions LinkedIn, Facebook and Twitter, which appear in four different business areas, namely Services, Suppliers, Technological Surveillance and Cost Reduction. This of course influences the number of recommendations received by these functionalities, however, it is not less significant that they are placed logically within different business areas so they deserve to be recommended more often than others. Thus, when a group of tools is involved in more than one business area, it is identified with a number in brackets corresponding to that number of areas. The specific total figures do not provide meaningful information, even less for this pilot study, nonetheless the appearance or absence of some specific tools in this top ten provides valuable information. To such effects, those tools which provide a solution to videoconference functionality can be found at the very top, these are tools designed to facilitate collaboration between employees and clients and to reduce communication costs, such as Google Hangout and Skype, among others. This kind of functionality is applicable to different business areas such as Internationalization, Suppliers, Human Resources, Costs Reduction and Administration and Bank Institutions and has generated high interest among participants. Together with these tools, the tools which provide the solution to subscribe to different social networks are ranked in second place. These go hand in hand with those used to manage subscriptions, such as Hootsuite and TweetDeck, ranked fourth. There is no doubt that despite these tools not being pure cloud computing technologies, they are the primary drivers of the incorporation of current enterprises into the cloud. This further demonstrates an increase of business interest in being aware of online reputation.

It is worth mentioning that the fifth and the sixth positions are occupied by tools aimed at solving accounting tasks, such as eFactura Online and Enneto for Online Billing functionality and Contabilidad Online and Cuentica for Online Accounting and invoicing. These solutions are proposed within Clients, Suppliers and Cost Reduction areas, and take advantage of one of cloud computing’s main characteristics, accessibility of different agents from different places. The collaboration between employees, suppliers and clients is based on the functionalities Collaborative Platforms which provide real-time collaboration through web conferencing and Content Management Systems which allow content to be published, edited and modified from a central interface. This is a pure example of cloud computing technology and it is well placed in the seventh position with specific tools such as MakeaNet.com and WordPress. The Feed Readers functionality solutions allow the companies to be aware of competitors’ actions, even to perform self-assessments. This activity has become an unavoidable task for current enterprises and it is well covered by cloud computing. These tools have generated enough interest to be placed in the eighth position, with solutions such as Google Reader (which stopped working in July 2013) and Netvibes. The technology surveillance tools such as Intelek and Litebi are included within the Services and Technology Surveillance areas in the diagnosis, providing decision making functionality; even if the importance of technology surveillance is not yet well-known by the majority of the enterprises, most of the participants have expressed interest in this functionality, as a result of which it appears in the top ten, so an increase in demand for such tools can expected. Finally the tenth most demanded functionality has been Online Project Management, with the eProwin Project tool. Even if it is only offered within the Human Resources and Cost Reduction business areas it has been one of the most demanded functionalities. In fact, the project management task requires a continuous updating and this probably needs to be done by various agents; cloud computing provides a satisfactory solution to that.

**Fig 15:**
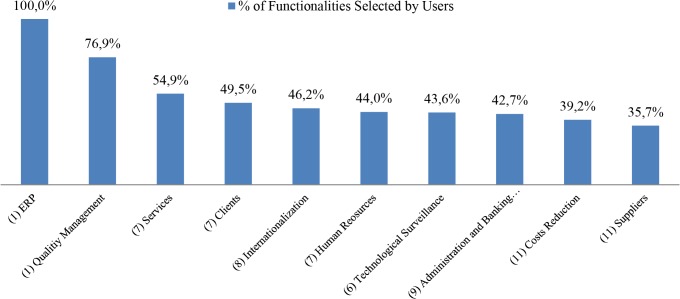
Business areas ranked by percentage of selection of its functionalities. The chart shows the business areas analyzed by the tool, arranged in terms of percentage of selection of the functionalities pertaining to each business area. In brackets the number of functionalities belonging to each business area.

Functionalities and tool analysis gives us some deeper insights. Each business area has its own functionalities, for instance the Services area is described through seven functionalities whereas Internationalization does it through eight. In this regard, it is interesting to show which areas have gained greater interest in terms of the average percentage of functionalities selected by the participants in each area, which can be seen in Fig 15.

The percentage gives us a good measure about how the participants have reacted to the different areas; in fact, a high percentage means that most of its functionalities have attracted the interest of the participants, whereas a low percentage means the opposite. It is worth checking the differences between the preliminary priority levels (shown in Fig 9), which are gathered before describing the functionalities themselves, against the percentage of selection described in this figure. The most surprising case is that of Quality Management, which was placed in last position in terms of interest but ranked second in percentage of selection. This is partially explained by the fact that it only has one functionality: Online Automation of Quality Management, nonetheless it is remarkable that even though the participants were not initially interested in it, once the functionalities are described they feel at least curious, behavior which can be extrapolated to cloud computing as a whole. In addition to this, as seen in Fig 15, the ERP’s functionality is selected by all the participants, it was well placed within the preliminary priority levels and none of the participants miss the opportunity to find out about the tool related to it. Most of the others are placed approximately in the same position in both figures, with the exception of Cost Reduction and Suppliers, which were placed third and fourth in Fig 9, yet in contrast they occupy the last positions in Fig 15. Both of them are described with 11 functionalities, a number apparently too large for the participants, as a result of which the percentage of selection was fewer than 40%. In conclusion, even if a business area arouses interest, enterprises are not ready to handle so many different tools, at most they select three or four. When analyzing these percentages, bear in mind that these only deals with advice about the tools, so when it comes to discuss adopting the tools, the percentages will be smaller.

**Fig 16:**
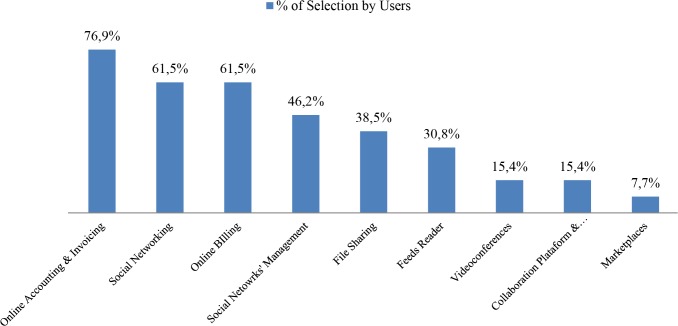
Ranking of functionalities and tools, Suppliers business area. The chart shows the functionalities and its tools belonging to the Suppliers business area, arranged by the percentage of selection by the pilot study participants.

**Fig 17:**
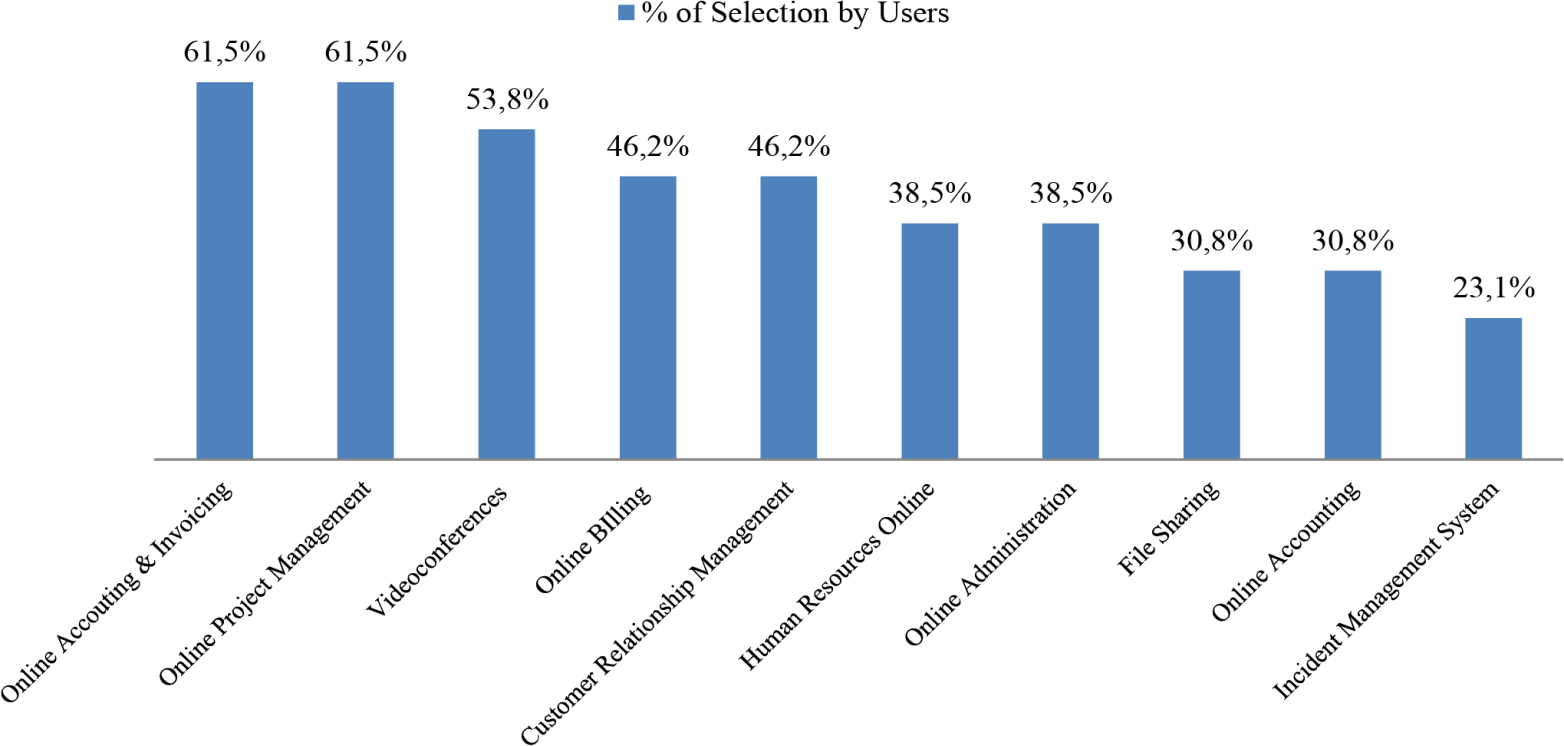
Ranking of functionalities and tools, Cost Reduction business area. The chart shows the functionalities and its tools belonging to the Cost Reduction business area, arranged by the percentage of selection by the pilot study participants.

Finally, the pilot study provides an opportunity to perform a further breakdown in order to identify the preferred functionalities and the corresponding tools for each business area. The following figures show two examples (Figs 16 and 17) for two different business areas. In the case of Suppliers, the participants have mostly selected the Online Accounting & Invoicing tools such as Cuentica, a traditionally cumbersome task which can be integrated and simplified through cloud computing. On the other hand the Marketplace functionality has attracted little interest, perhaps too ambitious a tool. In the case of the Cost Reduction area, Online Accounting & Invoicing is once again at the top, a clear interesting solution for the participants wherever it appears. In this case the Incident Management System closes the list, a tool most related to those businesses with strong after-sales services.

## Conclusions

Although, it could be argued that this is now a reality, it is also a fact that a large number of companies, especially SMEs, are not yet aware of either the existence of cloud technology or its benefits for the business sector. Therefore, it is necessary to implement mechanisms to overcome these problems which can be considered as barriers for cloud adoption. Consequently, a cloud computing adoption decision tool has been created, the main aim of which is to show the different SaaS applications available on the market and how these applications can enhance and manage business priority areas. Via the pilot study performed with different kinds of SMEs at local level, some interesting results have been established. Participants’ initial preferences were upgraded once the functionalities were explained; the fear of the unknown is a clear barrier which has to be overcome. There are certain areas more likely to boom in cloud computing, at least initially, whereas other areas need longer to get the market, as discussed in the results section. In this sense, an aid to all stakeholders involved could be an endeavor as with Bildosola et al. [43], where a methodology is presented to depict the cloud computing technology evolution path. In addition to this, each enterprise should recognize where its own path to the cloud is, its own Cloud Road.

The newcomers to the cloud should take into account several factors. From one side it is clear that SaaS applications are deployed in a cross-cutting manner in the whole company rather than in particular areas. Moreover, Commercial and Sales areas along with others such as Productive aspects, Finance Management, Quality, Human Resources and Innovation can be emphasized. On the other hand, the SaaS market is still in its infancy in many countries, so there are many bogus solutions within the cloud concept which create confusion in the market. It is important to remember that SaaS service offerings do not usually mention the migration process, which should be taken into account given its difficulty. Thus it must be analyzed carefully.

It can be concluded by saying that cloud computing is especially beneficial for startup companies, SMEs, entrepreneurs and companies that need to make new investments or do not have a stable infrastructure. For these companies this technology leads to a significant saving, in addition to increasing flexibility and competitiveness. This work is a try to help these actors to get into the cloud.

## Supporting Information

S1 Fig Decision Tree. The figure shows all the variables and conditions used to construct the decision tree. The decision tool is based on this decision tree and as a result of the combination of these variables and conditions different tools are proposed to the user.
(PDF)
