# Supplementary figures and images for: Design and Implementation of a Cloud Computing Adoption Decision Tool: Generating a Cloud Road
Source: PLoS One. 2015 Jul 31;10(7):e0134563. doi: 10.1371/journal.pone.0134563 (PMC4521817; doi:10.1371/journal.pone.0134563)

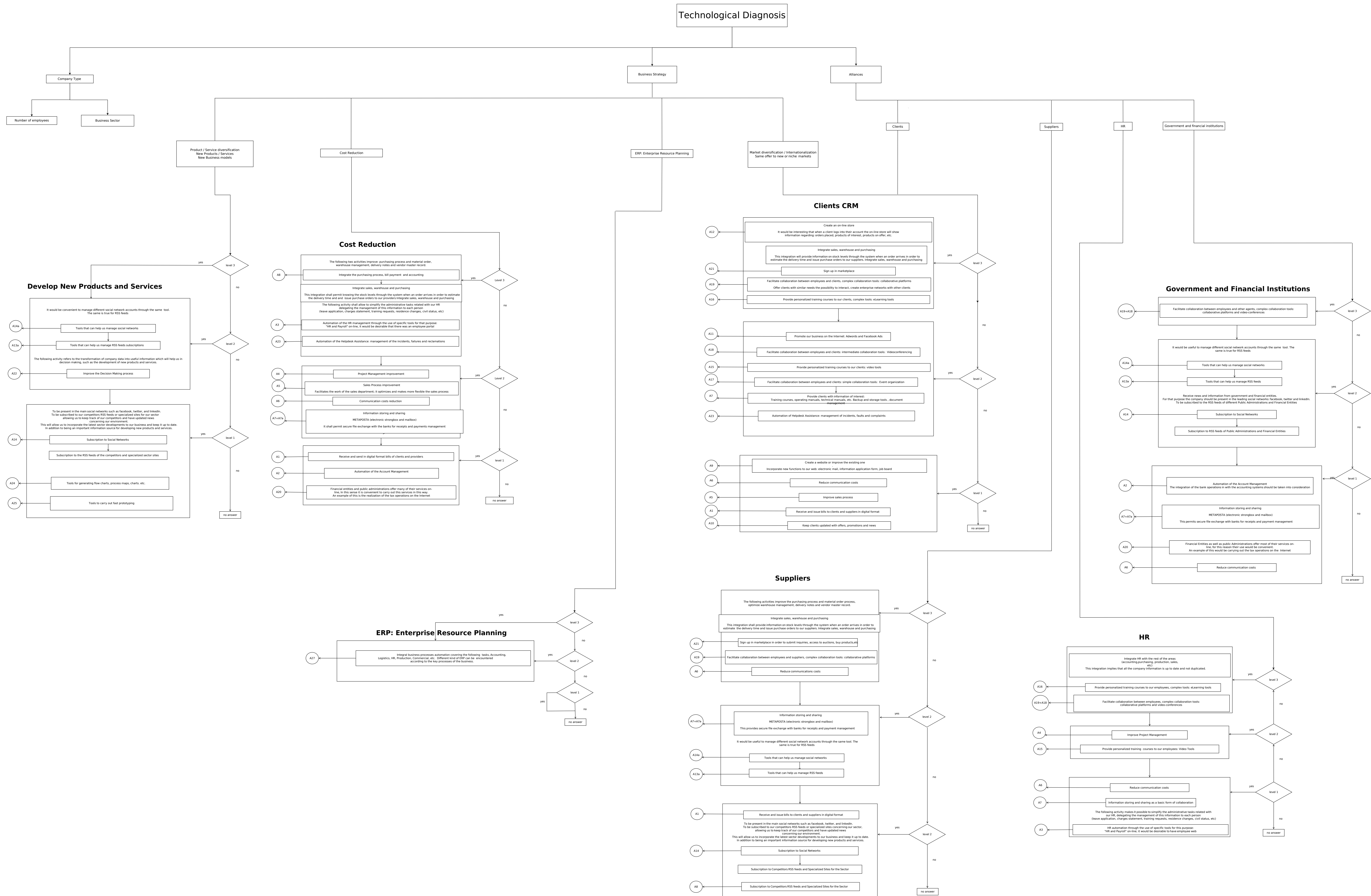

Supplement: S1 Fig — The figure shows all the variables and conditions used to construct the decision tree. The decision tool is based on this decision tree and as a result of the combination of these variables and conditions different tools are proposed to the user. (PDF) [file pone.0134563.s001.pdf]
